# MICA immune complex formed with alpha 3 domain-specific antibody activates human NK cells in a Fc-dependent manner

**DOI:** 10.1186/s40425-019-0687-9

**Published:** 2019-08-06

**Authors:** Changchun Du, Jack Bevers, Ryan Cook, T. Noelle Lombana, Kamalakannan Rajasekaran, Marissa Matsumoto, Christoph Spiess, Jeong M. Kim, Zhengmao Ye

**Affiliations:** 1 Department of Biochemical and Cellular Pharmacology, Genentech Inc, 1 DNA Way, South San Francisco, CA 94080 USA; 2 Antibody Engineering, Genentech Inc, 1 DNA Way, South San Francisco, CA 94080 USA; 3 Structural Biology, Genentech Inc, 1 DNA Way, South San Francisco, CA 94080 USA; 4 Cancer Immunology, Genentech Inc, 1 DNA Way, South San Francisco, CA 94080 USA; 5 Present address: NGM Biopharmaceuticals, 333 Oyster Point Blvd, South San Francisco, CA 94080 USA

**Keywords:** MICA, NKG2D, Immune suppression, Anti-MICA, Immunotherapy

## Abstract

**Background:**

One of the mechanisms by which tumors evade immune surveillance is through shedding of the major histocompatibility complex (MHC) class I chain-related protein A and B (MICA/B) from their cell surface. MICA/B are ligands for the activating receptor NKG2D on NK and CD8 T cells. This shedding reduces cell surface levels of MICA/B and impairs NKG2D recognition. Shed MICA/B can also mask NKG2D receptor and is thought to induce NKG2D internalization, further compromising immune surveillance by NK cells.

**Methods:**

We isolated human primary NK cells from normal donors and tested the suppressive activity of soluble recombinant MICA in vitro. Utilizing a panel of novel anti-MICA antibodies, we further examined the stimulatory activities of anti-MICA antibodies that reversed the suppressive effects of soluble MICA.

**Results:**

We show that suppressive effects of soluble MICA (sMICA) on NK cell cytolytic activity was not due to the down-regulation of cell surface NKG2D. In the presence of an α3 domain-specific MICA antibody, which did not obstruct NKG2D binding, sMICA-mediated NK cell suppression was completely reversed. Reversal of NK cell inhibition by sMICA was mediated by immune complex formation that agonized NKG2D signaling. Furthermore, this restorative activity was dependent on antibody Fc effector function as the introduction of Fc mutations to abrogate Fc receptor binding failed to reverse sMICA-mediated NK cell suppression. Furthermore, MICA immune complexes preformed with an α3 domain-specific antibody (containing a wild-type Fc) induced IFN-γ and TNF-α secretion by NK cells in the absence of cancer cells, whereas MICA immune complexes preformed with the Fc effectorless antibody failed to induce IFN-γ and TNF-α secretion. Finally, we demonstrated that MICA immune complexes formed with the α3 domain-specific antibody activates NKG2D on NK cells leading to the release of IFN-γ.

**Conclusions:**

Our results demonstrate that an α3 domain-specific MICA antibody can circumvent sMICA-mediated suppression of NK cell cytolytic activity. Moreover, our data suggest that MICA immune complexes formed with α3-specific antibodies can activate NKG2D receptor and restore NK cell function in a Fc-dependent manner. The clinical utility of α3 domain-specific MICA/B antibodies may hold great promise as a new strategy for cancer immunotherapy.

**Electronic supplementary material:**

The online version of this article (10.1186/s40425-019-0687-9) contains supplementary material, which is available to authorized users.

## Background

Natural killer (NK) cells are an important immune cell population contributing to anti-viral and anti-tumor immune responses [1]. Their activity is tightly regulated by a battery of stimulatory and inhibitory receptors. Natural killer group 2-member D (NKG2D) is one of the well-characterized activating receptors [2]. NKG2D is a type II transmembrane, homo-dimeric receptor expressed on the surface of almost all human NK cells, CD8 αβ^+^ T cells, γδ T cells, and NKT cells. Ligand engagement of the NKG2D receptor triggers a potent intracellular signaling cascade via the adaptor DAP10, leading to cytokine secretion and cytolysis of target cells [3].

A host of NKG2D receptor ligands have been identified, including the MHC class I chain related molecules A and B (MICA/B) [4] and the HCMV glycoprotein UL16-binding protein family molecules (ULBPs) [5]. MICA and MICB are cell surface glycoproteins encoded by two highly polymorphic genes, that reside in the human HLA class I locus. The extracellular portion of MICA/B contains a tripartite domain arrangement with membrane distal α1/α2 domains interacting with NKG2D and a membrane proximal α3 domain [6]. The expression of MICA/B is absent on most normal tissues, but is strongly induced by cellular stress conditions, including viral infections and cellular transformation due to DNA damage [7, 8]. The role of the MICA/B-NKG2D signal axis in tumor immune surveillance has been well documented [9]. In humans, NKG2D engagement by cell membrane-bound MICA/B has been shown to activate NK cells, γδ T cells and co-stimulate CD8 αβ ^+^ T cells in vitro [8]. In mice, tumor cells engineered to ectopically express murine NKG2D ligands Rae1b or H60 are dramatically rejected in immune competent mice via NK cell and CD8 T cell mediated mechanisms [10]. Conversely, spontaneous tumor development in genetically engineered mouse models of prostate cancer and B cell lymphomas are accelerated in NKG2D-deficient mice [11], reflecting the critical role of the NKG2D pathway in cancer immunosurveillance.

To escape NKG2D-mediated immune surveillance, it is hypothesized that tumors proteolytically shed MICA/B [12, 13] from the cell surface. In support of this notion, shed MICA/B can be found in sera of patients with many different cancer types, including prostate [14], colon [15], pancreatic carcinoma [16] and multiple myeloma [17]. Shed MICA/B has been hypothesized to dampen the host immune response mainly by inducing the down-regulation of cell surface NKG2D and destabilizing CD3ζ in the TCR/CD3 complex on CD8 T cells [18]. Given the mounting evidence of shed MICA/B in immune suppression, MICA/B is currently being investigated as a potential target for cancer immunotherapy. As the first hint of clinical relevance, Jiushi et al. reported that a melanoma patient receiving a combination therapy of anti-CTLA-4 antibody and autologous tumor cell vaccine secreting GM-CSF developed auto-antibodies against shed MICA accompanied by a reduction of serum MICA levels [19]. Furthermore, treatment-induced anti-MICA antibodies were demonstrated to reverse in vitro suppression of NK cells induced by soluble MICA. In addition, it has been reported that administration of a non-blocking monoclonal antibody specific to shed MICA/B along with an anti-CTLA-4 antibody synergistically boosts anti-tumor immune response and alleviates anti-CTLA-4 induced colitis in a genetically engineered model of spontaneous prostate cancer, TRAMP (Transgenic adenocarcinoma of the mouse prostate) when bred onto a MICA transgenic background [20]. Recently Ferrari de Andrade et al. showed that antibodies specific to the α3 domain of MICA block MICA/B shedding, thereby restoring cell surface MICA/B expression in vitro and impairing the growth of murine syngeneic tumors over-expressing full-length MICA in a NK cell-dependent fashion [21].

Although accumulating evidence points to the therapeutic potential of anti-MICA antibodies in preclinical animal models, the underlying mechanism of anti-MICA antibodies remains poorly characterized. In the current study, we examined the biological impact of anti-MICA antibodies in the presence of immunosuppressive soluble MIC proteins. We demonstrate that soluble MICA forms complexes with an α3 domain-specific anti-MICA antibody. Anti-MICA immune complexes reversed the immunosuppressive activities of soluble MICA by activating NKG2D through a Fc receptor-dependent mechanism. Accordingly, preformed anti-MICA immune complexes containing wild-type Fc effector function induced IFN-γ and TNF-α secretion by NK cells in the absence of tumor cells. Our study reveals a potential therapeutic mechanism of anti-MICA/B antibodies in the clinical setting. The clinical utility of therapeutic α3 domain-specific MICA/B antibody may hold great promise as a new strategy for cancer immunotherapy.

## Methods

### Cell lines

HMy2.C1R (referred as C1R) (ATCC CRL-1993), a human B lymphoblast cell line, was transfected with the coding sequence of MICA*002 allele and used as target cells. Parental C1R or MICA*002-expressing C1R (C1R-MICA*002) and primary human NK cells were cultured in RPMI-1640 media supplemented with 10% fetal bovine serum (FBS) (Thermo Fisher Scientific), 50 U/mL penicillin, 50 μg/mL streptomycin (Life Technologies), 2 mM glutamine (Thermo Fisher Scientific) and 1x non-essential amino acids (Thermo Fisher Scientific), and 14.3 mM β-mercaptoethanol (Sigma).

### Recombinant proteins

MICA*002 extracellular domain (MICA-ECD) was expressed and purified as previously described [22]. Recombinant human NKG2D-Fc, and TGF-β1 were purchased from R&D Systems. Goat anti-human IgG Fcγ fragment specific antibody was obtained from Jackson Immuno Research.

### Antibodies and cytokines

For FACS analysis anti-NKG2D antibodies (clones 5C6 (rat IgG2b) and clone 1D11(mouse IgG1)), rat IgG2b isotype control, mouse IgG1 isotype control, recombinant human IL-2, anti-human Fc, and anti-mouse IgG Fc were obtained from (eBioscience), and human Fc block from BioLegend. Anti-human CD56 allophycocyanin (APC) (clone HCD56) and 7-Aminoactinomycin D (7-AAD) were purchased from BD Biosciences. Unlabeled mouse anti-human MICA (clone AMO1, mouse IgG1) was acquired from MBL International. Mouse anti-MICA antibodies (clones 5E10, 7G10 and 6E1) were generated by immunizing BALB/c mice as described [22], and formatted to human IgG1 chimeras (wild-type Fc and N297G effector-less). Human IgG1 control antibody and mouse anti-human NKG2D antibody (clone 26F3, mouse IgG1) were generated at Genentech.

### Primary human NK cells

Peripheral blood samples were collected from Genentech healthy donor program. All of the blood donation procedures, recruitment materials, and forms were reviewed and approved by Genentech institutional review board. To isolate primary human NK cells, peripheral blood mononuclear cells (PBMC) were first isolated from the blood samples of healthy donors by density gradient centrifugation using Ficoll-Paque PLUS media (GE Health Care), and fresh NK cells were isolated by negative selection using NK cell isolation kit II (Miltenyi Biotec). NKG2D expression on NK cells was detected by anti-NKG2D (1D11) using FACSCalibur (BD Biosciences), and data were analyzed by FlowJo v10 (Tree Star). For NK cell cytolytic experiments, fresh NK cells were used immediately after isolation; for NKG2D down-regulation experiments, NK cells were cultured in the presence of 10 ng/mL IL-2 at 37 °C with 5% CO_2_ for 24 h.

### NK cytolytic activity assay

Parental C1R and C1R-MICA*002 cells were first washed with RPMI-1640 media. Fresh NK cells were co-cultured with the parental C1R or C1R-MICA*002 cells (target cells) at 10 to 1 ratio at 37 °C with 5% CO_2_ for 4 h.

To investigate whether soluble MICA suppresses NK cell function, NK cells were pre-incubated with 5 μM MICA-ECD at 4 °C for 4 h, followed by co-culture with target cells for 4 h. To assess target cell killing, co-cultured cells were harvested and blocked with human FcR block, followed by staining with 7-AAD and anti-CD56–APC in PBS/2 mM EDTA/0.5%FBS, and the samples of different treatments were analyzed by flow cytometry. C1R or C1R-MICA*002 cells were identified as CD56^−^ populations; NK cytolytic activity was defined as the frequency of 7AAD^+^CD56^−^ in the CD56^−^ target cell population. To investigate the impact of anti-MICA antibody treatment on the suppressive activity of MICA-ECD, anti-MICA antibody (human IgG1 clones 5E10, 7G10 and 6E1) were added at 2.5 μM to the NK and C1R-MICA*002 cell co-cultures. To preform MICA-ECD immune complex (MICA-IC), MICA-ECD and anti-MICA antibody were mixed at 2 to 1 M ratio in complete RPMI-1640 medium, and incubated at 37 °C for 30 min. To address whether Fc effector function was required for MICA immune complex-mediated NK killing activity, hIgG1 wildtype and N297G mutant forms of MICA antibody (clone 6E1) were used.

### NKG2D down-regulation assay

Fresh human NK cells were incubated with MICA-ECD (5 μM) or TGF-β1 (2 ng/mL) in the presence of 10 ng/mL IL-2 for 24 h at 37 °C with 5% CO_2_. NK cells were harvested and pre-incubated with human Fc block, followed by staining with an anti-NKG2D antibody (26F3, mouse IgG1); anti-NKG2D binding was detected by anti-mouse IgG secondary antibody and NKG2D expression was compared among all treatment conditions. To identify an anti-NKG2D antibody that does not compete with sMICA for NKG2D binding, NK cells were pre-incubated with or without 5 μM MICA-ECD, followed by anti-NKG2D PE (5C6 or 1D11) or anti-NKG2D mIgG1 (26F3) binding and detected by anti-mouse IgG Fc PE.

### Anti-MICA mAb and hNKG2D-Fc binding competition assay

To investigate whether anti-MICA mAbs (5E10, 7G10 and 6E1) compete with NKG2D for membrane-bound MICA binding, C1R-MICA*002 cells were first treated with Fc block and then incubated with 5 μg/mL human NKG2D-Fc in the presence of increasing amounts of anti-MICA antibodies for 30 min at 4 °C. Human NKG2D-Fc binding was detected by anti-human Fc secondary antibody by flow cytometric analysis.

### Tumor-free NK cell activation assay

MICA-immune complex (MICA-IC) was formed by mixing MICA-ECD with anti-MICA 6E1 (human IgG1 wildtype or Fc mutant N297G) (5 μM MICA-ECD and 2.5 μM 6E1) in complete RPMI1640 media, at 37 °C for 30 min. Fresh human NK cells were added (100,000 cells/well) and incubated with preformed-MICA-IC in the presence of 10 ng/mL IL-2. Supernatant samples were harvested at day 6 for IFN-γ and TNF-α release analyzed using Luminex xMAP platform (Thermo Fisher Scientific).

For plate-bound MICA-IC stimulation, flat bottom 96-well tissue culture plates (Costar) were pre-coated with goat anti-human IgG Fcγ-specific Ab at 100 μg/mL in PBS over night at 4 °C. MICA-IC was generated as above, and serially diluted. Coated plates were washed with PBS, and MICA-IC and NK cells (100,000 cells/well) were added and cultured in the presence of 10 ng/mL IL-2. Supernatant samples were harvested for IFN-γ analysis at day 6 using Luminex xMAP platform (Thermo Fisher Scientific).

### Statistical analysis

Statistical analysis was performed using GraphPad Prism, *p*-values were generated by unpaired t test.

## Results

### Soluble MICA suppresses NK cell cytolytic activity

**Fig. 1:**
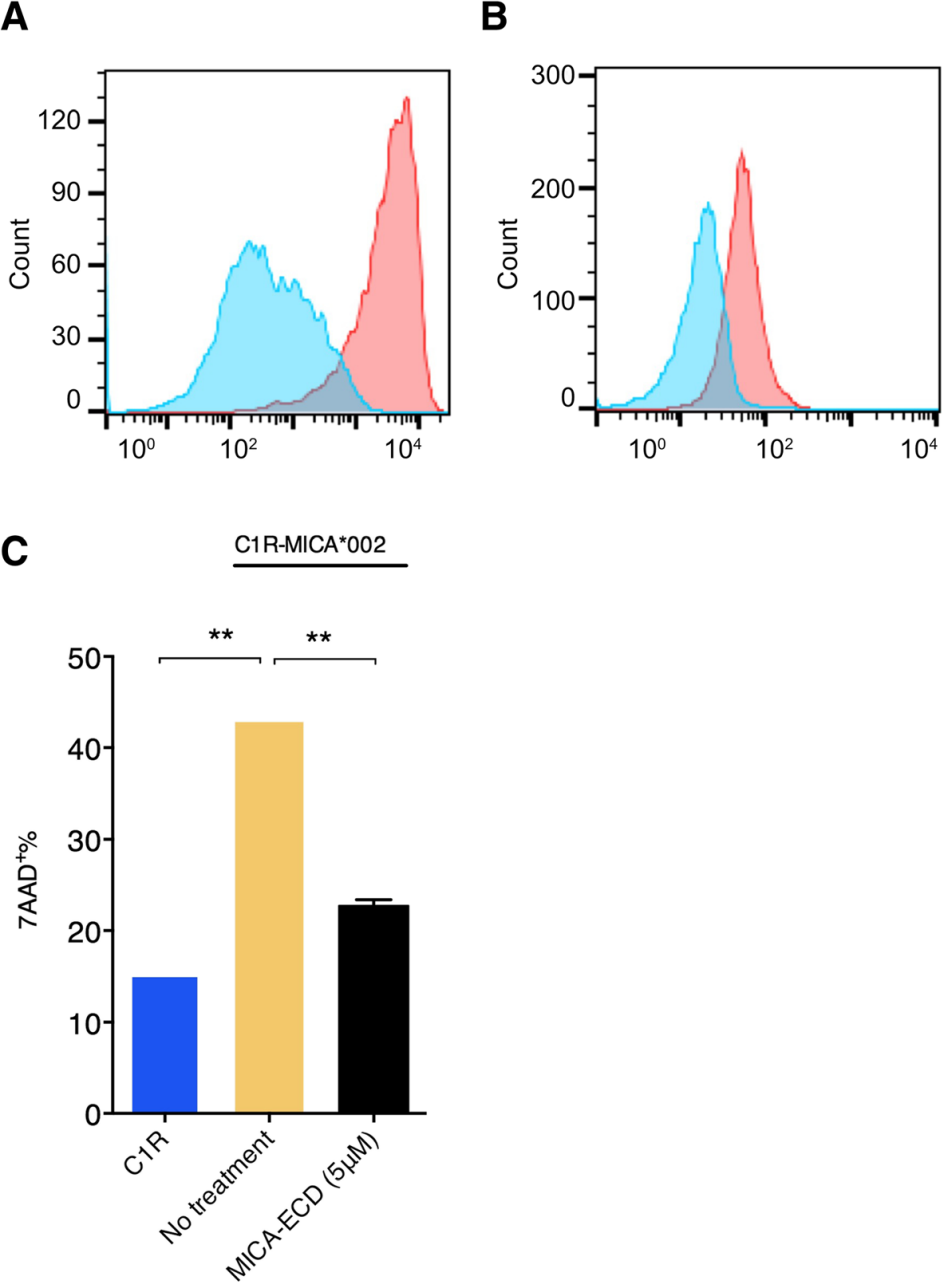
The suppressive effect of MICA-ECD on NK cell cytolytic activity. **a** The MICA*002 expression on the C1R-MICA*002 cell line was detected with a human NKG2D-Fc fusion protein, followed by the staining of a PE conjugated anti-Fc monoclonal antibody (red). An IL-23R-Fc protein staining was used as a specificity control (blue). **b** NKG2D receptor expression on primary human NK cells was detected by a PE-conjugated anti-NKG2D antibody (clone 1D11) (red), compared with isotype control antibody staining (blue). **c** The killing experiment of C1R-MICA*002 cells was conducted using NK cells in the presence or absence of recombinant MICA-ECD (5 μM) or no treatment. NK cell-mediated killing of the parental C1R cell line was shown as comparison. Each data point represents average of 2 technical replicates with error bar representing SEM, and the dataset is a representative of 3 independent experiments

Shed MICA/B-mediated impairment of NKG2D activation on NK and CD8 T cells has been well documented. In order to establish an in vitro cell culture system to examine the effects of sMICA on primary human NK cells, we generated a human C1R cell line that ectopically expressed full-length human MICA*002 (C1R-MICA*002). We chose the C1R human B lymphoblast cell line because it lacks the expression of endogenous MICA/B and is deficient for most MHC-I proteins. MICA*002 is a common MICA allele with approximately 27% prevalence in European-American population [23]. Cell surface expression of MICA on C1R-MICA*002 cells was confirmed by the binding of recombinant human NKG2D-Fc (Fig. 1a). Human NK cells were isolated from healthy donors and NKG2D expression was confirmed by staining with the anti-NKG2D antibody (clone 1D11) (Fig. 1b). To examine the effect of sMICA on NK cell cytolytic activity, fresh NK cells were co-cultured with C1R-MICA*002 cells for 4 h at a 10 to 1 effector to target ratio in the presence or absence of recombinant MICA-ECD (5 μM). In contrast to the parental C1R control, which induced approximately 15% target cell killing, C1R-MICA*002 cells induced approximately 40% cytolytic activity (Fig. 1c). Cytolytic activity was measured by examining 7-AAD uptake on target cells by flow cytometric analysis (Additional file 1: Figure S1). Augmented cytolytic activity induced by C1R-MICA*002 was presumably due to NKG2D receptor engagement on NK cells by MICA expressed on the cell surface of C1R cells. We then asked whether the addition of MICA-ECD suppresses NK cell mediated target killing. As expected, MICA-ECD reduced NK cell killing to a level comparable to that seen in the killing of parental C1R cell line (Fig. 1c). sMICA-mediated suppression of NK cell cytolytic activity supported the notion that shed MICA suppresses NKG2D-mediated NK cell killing.

### Soluble MICA does not down-regulate NKG2D expression on NK cells

**Fig. 2:**
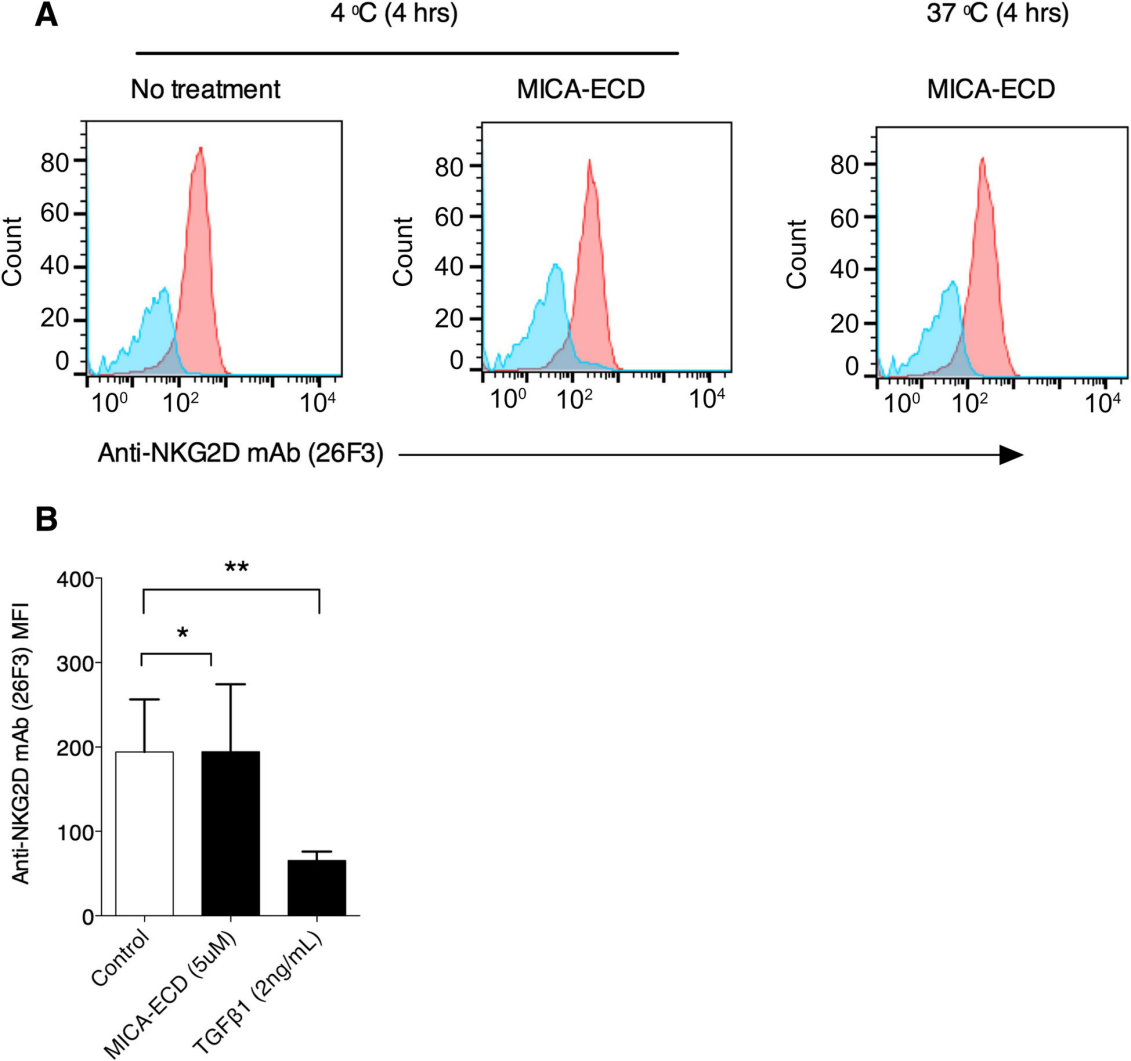
MICA-ECD does not down-regulate cell surface NKG2D receptor on NK cells. **a** Cell surface NKG2D expression on primary human NK cells was detected by an anti-NKG2D antibody (clone 26F3) (red) and isotype control antibody (blue) following the treatment of 5 μM MICA-ECD for 4 h at the indicated temperatures. **b** NKG2D expression on NK cells from three independent donors was detected by an anti-NKG2D antibody (clone 26F3) 24 h after treatment with 5 μM sMICA or 2 ng/mL TGF-β1. The NKG2D expression was indicated as the mean fluorescence intensity (MFI) of antibody stained cell population by flow cytometry analysis. The experiment was conducted using NK cells isolated from three independent donors (error bar representing SD), and the dataset is a representative of 2 independent experiments with *p*-values generated from unpaired t-test. *p > = 0.05; **p < 0.05

It has been suggested that sMICA induces NKG2D receptor internalization, leading to suppression of anti-tumor immunity [18]. To confirm whether sMICA induces NKG2D internalization, we incubated human NK cells with MICA-ECD and measured its effect on NKG2D cell surface expression. When NK cells were incubated with MICA-ECD (5 μM) at 4 °C for 4 h, a loss of NKG2D staining with two commercially available anti-NKG2D antibodies, clones 5C6 and 1D11 was observed (Additional file 2: Figure S2a and S2b), suggesting that the binding of these two anti-NKG2D antibodies was blocked by MICA-ECD. To investigate sMICA induced receptor internalization, we sought to identify anti-NKG2D antibodies that do not compete with sMICA for NKG2D binding. We generated a panel of anti-NKG2D antibodies and identified a non-competing anti-NKG2D antibody clone 26F3. Using 26F3 to stain cell surface NKG2D (Additional file 2: Figure S2c), we showed that NKG2D expression was not reduced after NK cells were incubated with MICA-ECD (5 μM) at either 4 °C or 37 °C for 4 h (Fig. 2a), suggesting that 26F3 and sMICA bind to different epitopes on NKG2D. Together, these results suggest that the utilization of commercially available anti-NKG2D clones may confound the analysis of NKG2D internalization by MICA treatment.

To confirm our observation that MICA-ECD treatment did not induce NKG2D down-regulation, we examined the effects of prolonged MICA-ECD exposure on NKG2D expression from three healthy donors using the non-competing anti-NKG2D antibody, 26F3. As shown in Fig. 2b and Additional file 3: Figure S3, NKG2D cell surface levels on NK cells from all three donors were not reduced after MICA-ECD treatment at 37 °C for 4 to 24 h. In contrast, NKG2D expression on NK cells was dramatically down-regulated 24 h after TGF-β1 treatment, a cytokine known to down-regulate the NKG2D receptor complex [24]. Together, our data demonstrate that sMICA does not induce NKG2D internalization on human primary NK cells in vitro. It remains a possibility that persistent exposure to membrane-bound MICA induces NKG2D down-regulation [25].

### An α3-specific anti-MICA antibody reverses soluble MICA-mediated NK cell suppression

**Fig. 3:**
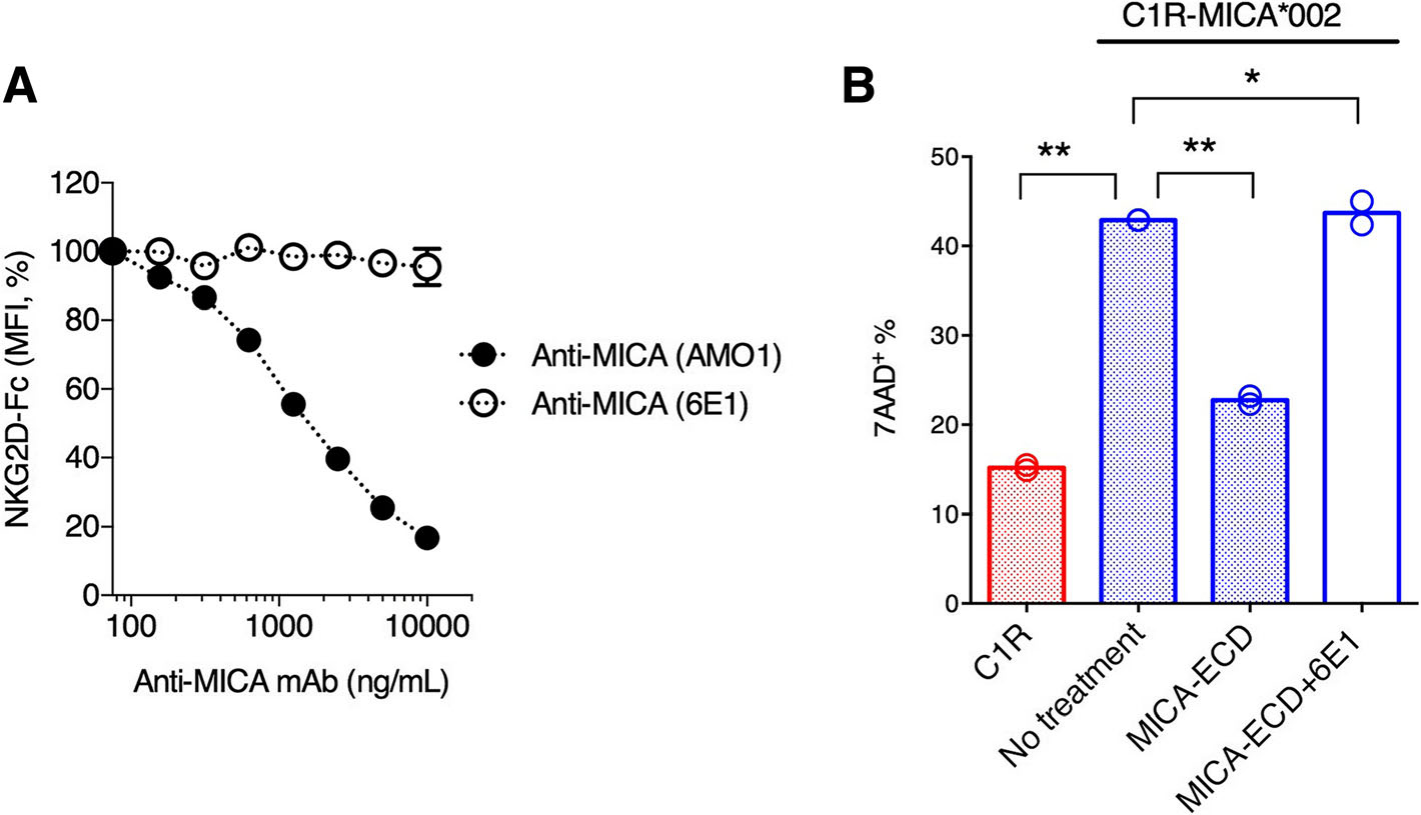
Suppressive effect of MICA-ECD on NK cytolytic activity is reversed by an anti-MICA antibody. **a** The binding of NKG2D-Fc fusion protein to the C1R-MICA*002 cell line was conducted in the presence of increasing amount of an anti-MICA antibody clone AMO1 (MICA α1α2-specific) or the anti-MICA/B mAb clone 6E1. The normalized mean fluorescence intensity (MFI, %) of NKG2D-Fc fusion protein binding is shown, each data point represents average of 2 technical replicates with error bar representing SEM, and the dataset is a representative of 2 independent experiments. **b** C1R-MICA*002 cell line killing was conducted side by side with C1R cells using human NK cells. NK cells were pretreated with MICA-ECD, MICA-ECD plus anti-MICA/B clone 6E1 or no treatment for MICA*002 cell line killing, and NK cell killing of parental C1R cell line was used for comparison. Each data point represents average of 2 technical replicates, and the dataset is a representative of 3 independent experiments with *p*-values generated from unpaired t-test. *p > = 0.05; **p < 0.05

Our results suggest that NK cell suppression occurs when sMICA masks the NKG2D receptor, blocking NKG2D engagement by cell surface MICA. To investigate the effect of anti-MICA antibodies on sMICA-mediated NK cell suppression, we generated a panel of anti-MICA/B antibodies and identified an α3 domain-specific antibody, clone 6E1, that does not block the MICA-NKG2D interaction (Additional file 7: Table S1). Like other members of the non-classical MHC-I family, MICA is comprised of plasma membrane distal α1 and α2 domains that participate in NKG2D binding and a membrane proximal α3 domain that does not interact with NKG2D [26]. As expected, the addition of an α1/α2 domain-specific antibody, AMO1, blocked the binding of recombinant human NKG2D-Fc fusion protein to MICA*002 C1R cells in a concentration-dependent manner (Fig. 3a). In contrast, the α3 domain specific antibody 6E1 did not interfere with the MICA-NKG2D interaction (Fig. 3a). When we tested the activity of anti-MICA antibodies in NK cell cytolytic assays, 6E1 treatment enhanced NK cell activity in the presence of MICA-ECD, and restored cytolytic activity to levels comparable to conditions without MICA-ECD treatment (Fig. 3b). This result revealed a new function of anti-MICA α3 domain-specific antibodies in reversing MICA-ECD-mediated NK cell suppression.

### α3-specific anti-MICA antibody reverses soluble MICA mediated NK cell suppression by agonizing NKG2D

**Fig. 4:**
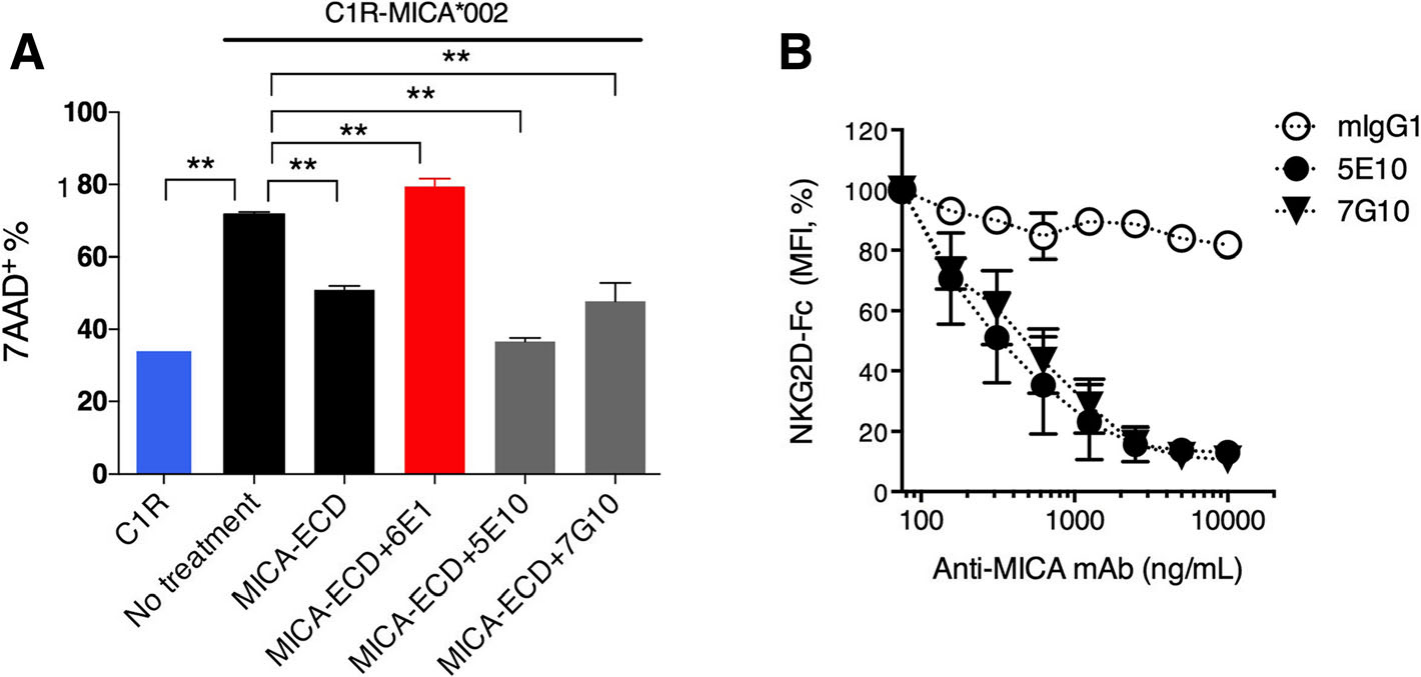
MICA/anti-MICA antibody immune complex restores NK cell killing in vitro. **a** C1R-MICA*002 cell killing was assessed by co-culturing C1R-MICA*002 cell line with primary NK cells. The NK cells were pretreated with MICA-ECD alone, MICA-ECD plus anti-MICA/B clones, 6E1 (MICA α3-specific) or 5E10 (MICA α1α2-specific) or 7G10 (MICA α1α2-specific) as preformed MICA-immune complexes, or no treatment. NK cell killing of parental C1R cell line was used for comparison. Each data point represents average of 2 technical replicates with error bar representing SEM, and the dataset is a representative of 3 independent experiments with *p* values generated from unpaired t test. **b** The binding of NKG2D Fc fusion protein to MICA on C1R-MICA*002 cell line was conducted in the presence of increasing amount of isotype antibody mIgG1, anti-MICA/B clone 5E10 or 7G10. The normalized MFI (%) of NKG2D-Fc fusion protein binding is shown, each data point represents average of 2 technical replicates with error bar representing SEM, and the dataset is a representative of 2 independent experiments. *p > = 0.05; **p < 0.05

Since the MICA α3 domain does not participate in the MICA-NKG2D interaction, we rationalized that anti-MICA clone 6E1 promotes resistant to MICA-ECD treatment through non-blocking mechanisms. For other soluble factors, complexing cytokines such as IL-2 or IL-15 to non-blocking antibodies have been shown to agonize receptor binding through the generation of a signaling competent immune complex [27, 28]. In this regard, we hypothesized that the non-blocking anti-MICA clone 6E1 is able to induce NKG2D signaling by forming an immune complex with soluble MICA. To test this hypothesis, we preformed MICA immune complexes (MICA-ICs) with 6E1, an α3 domain specific antibody and subsequently examined their ability to reverse sMICA-mediated suppression of NK cell killing. Interestingly, MICA-ICs preformed with the α3 domain-specific antibody 6E1 reversed MICA-ECD-mediated suppression of NK cell killing (Fig. 4a). Consistent with a role of enhanced cytolytic activity, MICA 6E1-ICs potentiated Granzyme B release (Additional file 4: Figure S4). o determine whether MICA-IC activity requires NKG2D binding, we compared MICA-ICs preformed with 6E1 to those preformed with α1/α2 domain-specific anti-MICA antibodies, 5E10 and 7G10(Additional file 7: Table S1). By binding to the α1/α2 domains of MICA, 5E10 and 7G10 blocked NKG2D binding (Fig. 4b). In contrast to MICA-ICs preformed with 6E1, MICA-ICs preformed with either 5E10 or 7G10 failed to reverse MICA-ECD-mediated suppression of NK cell killing activity or induce granzyme B release (Fig. 4a and Additional file 4: Figure S4). The key differential feature of these antibodies is that 6E1 does not interfere with MICA-NKG2D interaction because it binds to the α3 domain, which is distal to the NKG2D binding site on MICA, whereas antibodies 5E10 or 7G10 interfere with NKG2D binding. In this regard, antibodies 5E10 and 7G10 not only prevent MICA-ICs from engaging NKG2D but also block cell surface MICA-NKG2D interactions leading to the inhibition of the NKG2D signaling pathway. Alternatively, it can be hypothesized that α3-domain specific anti-MICA antibodies prevent MICA cleavage, increasing the density of MICA on the cell surface to enhance cytolysis. Although 6E1 treatment is sufficient to enhance MICA surface stabilization with prolonged exposure, we failed to detect increased MICA surface expression after 4 h of 6E1 treatment, mimicking the time point for NK cell cytolysis studies (Additional file 5: Figure S5). Collectively, our results suggest that preserving the MICA-NKG2D interaction is essential for the reversal of sMICA-mediated NK cell suppression by MICA-ICs. In addition to MICA-IC mediated NKG2D engagement, all three MICA antibodies (6E1, 5E10, 7G10) showed comparable ADCC activities to MICA expressing C1R cells in the absence of soluble MICA (Additional file 6: Figure S6). However, in the presence of high levels of soluble MICA, we speculate that ADCC effect plays a minor role because most of the therapeutic antibodies reside in MICA-ICs.

### MICA-ICs with α3 domain-specific antibody 6E1 activate NK cells in a Fc-dependent manner

**Fig. 5:**
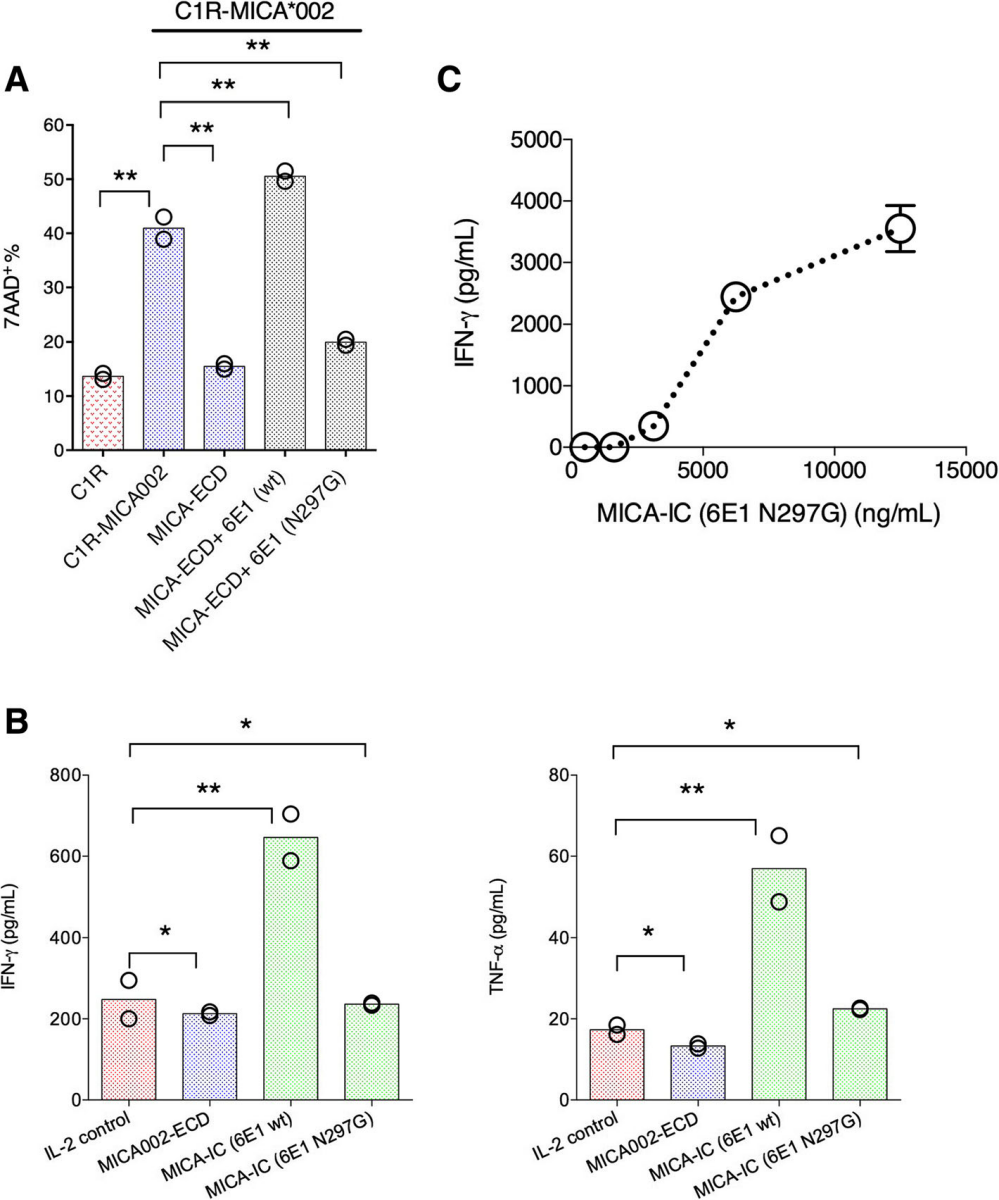
MICA immune complex formed with anti-MICA antibody clone 6E1 directly activates NK cells. **a** C1R-MICA*002 cell killing was assessed by co-culturing C1R-MICA*002 cell line with primary NK cells. The NK cells were pretreated with MICA-ECD alone, MICA-IC preformed with 6E1 (hIgG1, wildtype), MICA-IC preformed with 6E1 Fc effectorless mutant (hIgG1, N297G), or no treatment; NK cell killing of parental C1R cell line was used for comparison. Each data point represents the average of 2 technical replicates, the dataset is a representative of 3 independent experiments and *p* values were generated from unpaired t test. **b** NK cells were treated with MICA-ECD alone, MICA-IC preformed with 6E1 (hIgG1 wildtype) or MICA-IC preformed with 6E1 Fc effectorless mutant (hIgG1, N297G); IFN-γ and TNF-α secretion was analyzed using Luminex platform. Each data point represents average of 2 technical replicates, and the dataset is representative of 3 independent experiments and *p* values were generated from unpaired t test. **c** NK cells were cultured with MICA-IC preformed by 6E1 (hIgG1, N297G) that was bound to the goat anti-human Fc antibody coated to the surface of assay plate; IFN-γ and TNF-α secretion was analyzed using Luminex platform. Each data point represents average of 2 technical replicates (error bar represents SEM), and the dataset is a representative of 3 independent experiments. *p > = 0.05; **p < 0.05

To further explore the mechanism of MICA-IC-NKG2D signaling, we wanted to determine whether Fc effector function is required for MICA-ICs to restore NK cell killing activity. To this end, we generated a Fc effectorless mutant of 6E1 (hIgG1 N297G), in which Fc receptor binding is abolished. In our co-culture system, we found MICA-ICs formed with the 6E1 Fc effectorless mutant (hIgG1 Fc N297G) failed to reverse MICA-ECD-mediated inhibition of NK cell killing (Fig. 5a), indicating Fc receptor engagement is required for MICA-ICs to reverse sMICA-mediated NK cell suppression. We next asked whether the tethering MICA-ICs on Fc receptor-bearing NK cells is able to activate NK cells in the absence of tumor cells. Indeed, MICA-ICs (wild-type hIgG1 Fc) induced IFN-γ and TNF-α production by NK cells after 6 days in culture with IL-2 (Fig. 5b). In sharp contrast, IFN-γ and TNF-α induction was not observed with MICA-ICs formed with the Fc effectorless mutant of 6E1 (Fig. 5b). Because the effects of MICA-IC may be caused by Fc receptor agonism in addition to NGK2D activation, we next examined whether MICA-ICs can activate NKG2D on NK cells. To this end, we immobilized MICA-ICs preformed with the Fc effectorless 6E1 to the surface of a tissue culture plate via a secondary goat anti-human Fc antibody. We employed the Fc effectorless version of 6E1 with plate-bound stimulation to minimize the contribution of Fc receptor signaling. We found that these immobilized MICA-ICs preformed with Fc effectorless 6E1 was fully capable of inducing the IFN-γ secretion by NK cells (Fig. 5c). Altogether, our results demonstrate that MICA-ICs can activate NKG2D on NK cells and stimulate NK cell activities in a Fc-dependent manner. In summary, our results provide new insights on approaches to enhance NKG2D agonism in the presence of soluble MICA in cancer. Furthermore, our studies suggest a potential mechanism of action for α3-specific anti-MICA antibodies.

## Discussion

The induced expression of MICA/B by cellular stress such as viral infection and DNA damage in tumors facilitates immune surveillance. However, tumors developed an escape mechanism by shedding cell surface MICA/B. It was previously thought that shed MICA/B dampens NKG2D-dependent anti-tumor immunity by masking cell surface NKG2D receptor and inducing NKG2D down-regulation. In this report, using a novel NKG2D-specific antibody, we showed that surface NKG2D expression may not be downregulated by sMICA, suggesting alternative mechanisms of sMICA mediated suppression.

We confirmed that sMICA suppresses NK cell cytolytic activity. However, our results suggest that suppression is not due to sMICA-mediated NKG2D down-regulation on primary human NK cells. Using NK cells isolated from multiple normal human donors, we demonstrated that treating NK cells with a high level of sMICA for up to 24 h failed to induce NKG2D internalization when we used a novel non-competing NKG2D antibody as the detection antibody. However, when we used commercially available anti-NKG2D antibodies to detect NKG2D expression in the same setting, surface NKG2D level was reduced suggesting the binding epitope of these antibodies were preoccupied by sMICA. These data highlight the importance of using non-competing detection antibodies to assess ligand-induced receptor internalization. Additionally, our finding is consistent with the report that NKG2D down-regulation is primarily driven by the persistent exposure to cell membrane-bound MICA [25]. Because the binding affinity of MICA is relatively low (Kd: 0.5 to 1 μM) [29, 30], we chose a high amount of MICA-ECD to conduct in vitro NK cell suppression studies and to generate immune complexes for in vitro NK cell activation assays.

In our human NK and cancer cell co-culture system, we demonstrated that MICA-ICs formed with an α3 domain specific anti-MICA antibody can activate NKG2D and reverse sMICA-mediated NK cell suppression in a Fc-dependent fashion. Our results reveal a new strategy for cancer immunotherapy by harnessing the MICA/B-NKG2D signaling pathway, even in the presence of immunosuppressive soluble MICA, which is prevalent in multiple cancers. Traditional approaches to block soluble MICA binding limits the potential NKG2D signaling by also blocking surface MICA-NKG2D interactions. By targeting anti-MICA antibodies to the α3 domain of MICA, we were able to preserve the MICA-NKG2D interaction. The potential activities of MICA-ICs were most pronounced when a tumor-free NK cell system was used, indicating that cancer cell co-cultures may further impede immune cell activity (Fig. 4). Our data also show that Fc receptor binding by MICA-ICs is required for stimulatory activity, which parallels the activity of cytokine-immune complexes, as reported for IL-2 and IL-15 [27, 28]) (Fig. 5). In this regard, α3-domain specific antibody 6E1 supports the formation of a NKG2D agonistic immune complex. By coupling NKG2D recognition properties of sMICA with the signaling function of activating Fc gamma receptors, 6E1 immune complexes are able to overcome the inhibitory activity of soluble MIC proteins. Due to the high prevalence of soluble MIC proteins in multiple cancers, targeting the α3 domain serves as an attractive approach to enhance NKG2D signaling in cancers with elevated soluble MICA levels.

We have demonstrated that sMICA-IC activates NK cells by engaging the NKG2D receptor (Fig. 5). In addition to its intrinsic activity associated with NKG2D, MICA may also serve as a tumor-antigen for antibody-dependent cellular cytotoxicity (ADCC). As expected, all anti-MICA antibodies with intact Fc effector function mediated ADCC activity, regardless of epitope specificity (Additional file 6: Figure S6). Both α3-specific and α1/α2- specific anti-MICA antibodies induced comparable ADCC activities in the absence of exogenous sMICA administration. In cancer patients, MICA shedding is predicted to antagonize ADCC activity by serving as an antibody sink and reducing surface MICA expression. Therefore, antibodies specific for the α1 and α2 domains may have limited anti-tumor activity in the presence of soluble MICA. In contrast,, α3-specific anti-MICA antibodies have the potential to form NKG2D-engaging immune complexes with shed MICA/B, revealing a novel mechanism of action for anti-MICA antibody therapeutics.

In cancer cell lines, MICA cleavage has been mapped to the α3 and stalk domains [31]. Multiple metalloproteases harbor MICA cleavage activity, suggesting that inhibiting individual metalloproteases may not be sufficient to prevent MICA shedding [32]. However, antibodies that target the α3 domain are predicted to sterically impair metalloprotease access, thereby inhibiting MICA cleavage. In addition to forming MICA immune complexes, α3 domain-specific antibodies have the potential to enhance MICA density on cancer cells, and impair the release of shed MIC protein. In support of this, we show that an α3 domain-specific antibody 6E1 can stabilize surface MICA (Additional file 5: Figure S5). Cleavage inhibition was not unique to the C1R cell line, as multiple cell lines derived from different cancers and harboring different MICA alleles were also sensitive to α3 domain specific antibodies, including HCC1534, MEL-JUSO and SK-MEL cells (data not shown).

**Fig. 6:**
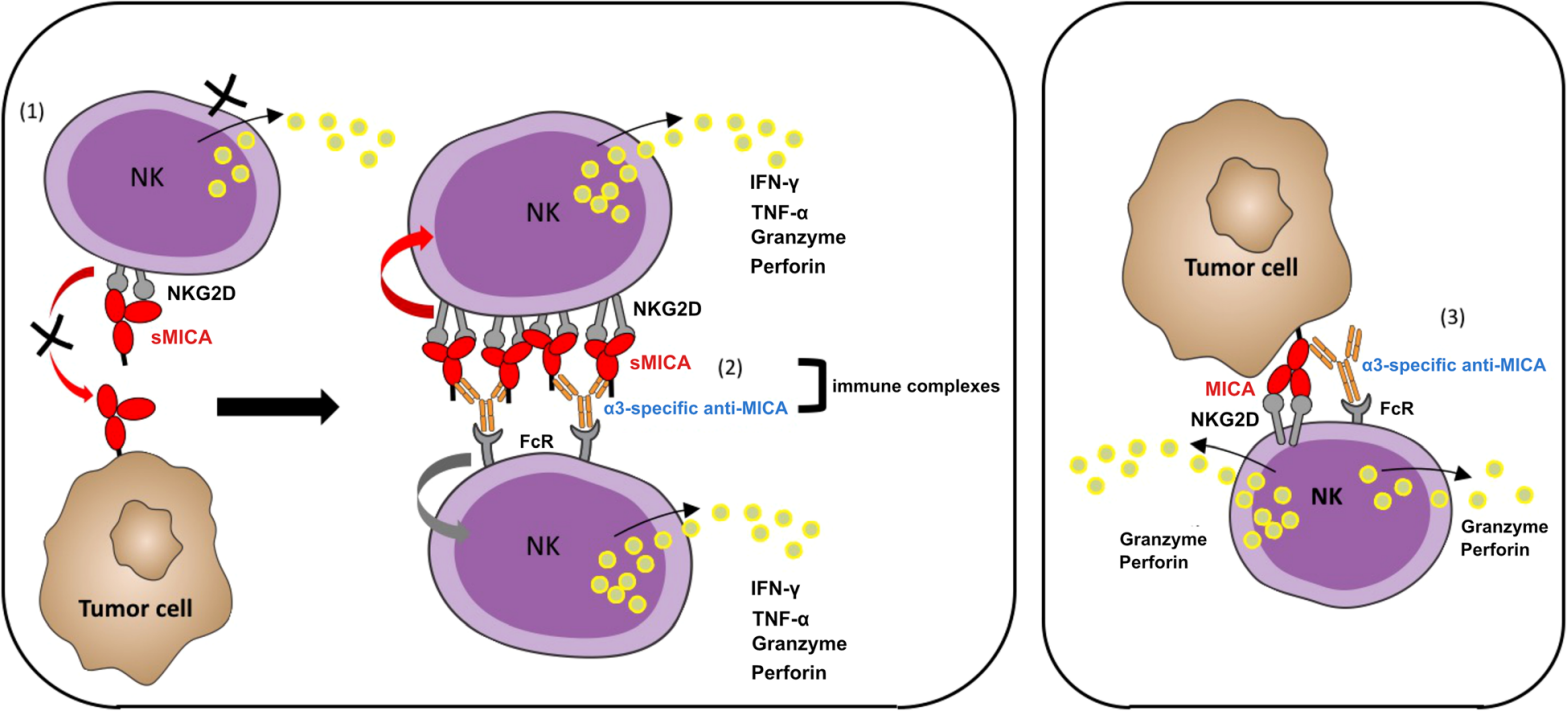
A working model of the action of MICA α3-specific antibody alone and of MICA immune complex. A working model is proposed to illustrate 1) shed MICA blocks NKG2D mediated cellular interactions with surface-bound MICA (left panel); and 2) MICA α3-specific antibody can form immune complexes with sMICA leading to NKG2D and Fc-dependent NK cell activation (left panel); 3) MICA α3-specific antibody induces ADCC-mediated tumor cell killing and augments NKG2D signaling by stabilizing tumor surface MICA (right panel)

The observation that MICA-ICs formed with α3 domain-specific antibodies agonized NKG2D in tumor-free conditions suggests that the agonistic properties of MICA-ICs is not unique to a particular tumor type (Fig. 5), and that MICA-ICs formed within a tumor mass can potentially activate NK-mediated anti-tumor responses. Based on our study, we propose a working model that illustrates how an α3 domain-specific MICA antibody can drive NK cell activation. α3 domain-specific antibodies have the potential to agonize NKG2D through MICA-ICs. They also can stabilize cell surface MICA, leading to enhanced NKG2D-mediated NK cell cytolytic and Fc gamma receptor-mediated ADCC activities (Fig. 6). In contrast, immune complexes formed by the α1/α2 domain-specific MICA antibodies cannot activate NKG2D signaling because such antibodies disrupt MICA-NKG2D interactions (Additional file 6: Figure S6). The relative contribution of these non-mutually exclusive mechanisms to the overall tumor killing activity requires further study. We speculate that, during the early stage of α3 domain-specific MICA antibody treatment, most of the anti-MICA antibody will be in MICA-immune complexes due to the high level of circulating MICA. Therefore, MICA-IC mediated NKG2D signaling will likely play a major role. As antibody treatment progresses, uncomplexed α3 domain specific MICA antibodies will be available to inhibit MICA shedding, leading to the reduction of sMICA and the restoration of cell surface MICA. At this stage, the cell surface NKG2D signaling together with Fc receptor mediated ADCC will likely play a dominant role. Also based on our working model, it can be hypothesized that MICA-IC induces NK cell fratricide by bridging activated NK cells in close proximity. However, in our co-culture system, we found that NK cell viability was not affected in the presence of MICA-ICs (Additional file 4: Figure S4c). It is also worth noting that our results were obtained using a tumor cell line that ectopically expresses MICA. Further studies using cancer cell lines that endogenously express and shed MICA will likely provide more insights for the proposed mechanism of action for α3 domain specific MICA antibodies. Overall, our data are consistent with a recent report showing that shedding inhibition mediated by an α3 domain-specific MICA antibody can drive NK cell activation in vitro and in vivo [21]. In addition, the uptake of MICA-ICs by macrophages and dendritic cells within the tumor mass can potentially enhance the priming of anti-tumor immune responses, and amplify the therapeutic activities of anti-tumor agents.

To further assess the role of MICA-ICs in vivo, tumor models in immune competent mice can be employed to test the ability of preformed MICA-ICs to stimulate anti-tumor immune responses. Due to the low binding affinity of MICA to NKG2D, it is conceivable that testing the activities of MICA/B immune complex using mouse models can be challenging. A recent study demonstrated that anti-MICA/B antibody enhanced NK cell-dependent destruction of tumor spheroids [33]. Such a 3D-model can be valuable tool for further testing the role of MICA-ICs and predicting the therapeutic activity in vivo. Future studies aimed to assess the anti-tumor activity of MICA specific antibodies may require complex mouse genetic engineering to incorporate a human transgene to minimize immunogenicity of human MIC proteins. Our results suggest that α3 domain specific anti-MICA antibodies possess the therapeutic potential to overcome immune suppression in tumors that shed high levels of MICA/B proteins.

## Conclusions

Using primary human NK cells and in vitro system, our studies highlight the significance of shed MICA-mediated immune suppression and further reveal the stimulatory activities of MICA immune complexes formed by α3 domain-specific MICA antibodies. We believe that our findings provide a promising strategy for developing novel biologics for cancer immunotherapy.

## Additional files

Additional file 1: **Figure S1.** FACS gating strategy for NK cytolytic activity assay. To analyze the NK cytolytic data acquired on FACSCalibur, target tumor cells were initially gated based on forward scatter (FSC) vs. side scatter (SSC). The CD56^−^ target cell population was subsequently gated based on the dot plot of CD56-APC vs. 7-AAD to exclude contaminating NK cells; the 7-AAD^+^ target cell was finally gated within the CD56^−^ gate to report target cell death as the percentage of 7-AAD^+^ cell population within the total CD56^−^ cell population. (JPG 204 kb)

Additional file 2: **Figure S2.** The anti-NKG2D mAb clone 26F3 does not compete with MICA-ECD. NKG2D on NK cells was not detected by anti-NKG2D clones 5C6 (A) or 1D11 (B), but by 26F3 (C) following treatment with 5 μM MICA-ECD at 4 °C for 4 h. The experiment was conducted using NK cells isolated from three independent donors (error bar representing SD), and *p*-values were generated from unpaired t-test. *p > = 0.05; **p < 0.05. (JPG 336 kb)

Additional file 3: **Figure S3.** TGFβ1 but not MICA-ECD down-regulates NKG2D on NK cells in the temporal analysis of NKG2D expression. NK cells from 3 healthy donors were treated with soluble MICA-ECD, TGFβ1 or no treatment, NKG2D expression was analyzed using non-competing anti-NKG2D antibody 26F3 on FACSCalibur at 4 (A), 8 (B) and 24 (C) hour time point. The NKG2D expression was indicated as the mean fluorescence intensity (MFI) of antibody stained cell population by flow cytometry analysis. The error bar represents SD, and the dataset is a representative of 2 independent experiments with *p*-values generated from unpaired t-test. *p > = 0.05; **p < 0.05. (JPG 227 kb)

Additional file 4: **Figure S4.** The MICA immune complexes formed with α3-specific antibody 6E1 reverse MICA-ECD-mediated NK cell suppression and induced Granzyme B release. a, C1R-MICA*002 cell line killing experiment was conducted by co-culturing C1R-MICA*002 cell line with primary NK cells for 4 h. The NK cells were pretreated with MICA-ECD alone, MICA-ECD plus anti-MICA/B clones, 6E1 (MICA α3-specific) or 5E10 (MICA α1α2-specific) or 7G10 (MICA α1α2-specific) as preformed MICA-immune complexes, or no treatment. Each data point represents an average of 3 technical replicates with error bar representing SD, and the dataset is a representative of 3 independent experiments with *p* values generated from unpaired t test. b, Granzyme B release in the supernatants of C1R-MICA*002 cell line killing experiment was quantified by ELISA assay (Human Granzyme B DuoSet ELISA kit, R&D systems), each data point represents average of 3 technical replicates with error bar representing SD, and the dataset is a representative of 3 independent experiments with *p*-values generated from unpaired t-test. c, NK cell viability in the co-culture across all experiment groups were examined and the percentage of CD56^+^ 7AAD^−^ NK cells in total NK cell population are shown. Each data point represents average of 3 technical replicates with error bar representing SD, and the dataset is a representative of 3 independent experiments with *p*-values generated from unpaired t-test. *p > = 0.05; **p < 0.05. (JPG 2307 kb)

Additional file 5: **Figure S5.** MICA α3-specific antibody 6E1 but not MICA-immune complexes stabilizes cell surface MICA. C1R-MICA*002 cells were treated by control hIgG1, 6E1, 5E10, 7G10 or MICA immune complex preformed with each of the 3 MICA antibodies, and the MICA expression was captured by non-competing α1α2-specific anti-MICA antibody (clone 6D4, eBioscience). The samples were collected at 4 (A) and 8 (B) hour time point, each data point is an average of 3 technical replicates with error bar representing SD, and the dataset is a representative of 2 independent experiments with *p*-values generated from unpaired t-test. *p > = 0.05; **p < 0.05. (JPG 209 kb)

Additional file 6: **Figure S6.** MICA antibodies induce ADCC. C1R-MICA*002 cell line ADCC experiment was conducted by co-culturing C1R-MICA*002 cell line with primary NK cells (10 to 1 effector to target ratio) in the presence of 10 μg/mL control hIgG1 or anti-MICA antibodies, 6E1, 5E10 or 7G10 for 4 h, the data is the average of 3 technical replicates with error bar representing SD, and the dataset is a representative of 3 independent experiments with *p*-values generated from unpaired t-test. *p > = 0.05; **p < 0.05. (JPG 95 kb)

Additional file 7: **Table S1.** Specificity and affinity of anti-MICA antibodies (DOCX 14 kb)

## Data Availability

All data supporting the conclusion of this study has been included within the article.

## Abbreviations

ADCC: Antibody-dependent cell-mediated cytotoxicity
ECD: Extracellular domain protein
IC: Immune complex
IFN: Interferon
mAb: Monoclonal antibody
MICA/B: MHC class I chain related molecules A and B
NK cells: Natural killer cells
NKG2D: Natural killer group 2-member D
sMICA: Soluble MICA
TNF: Tumor necrosis factor
TRAMP: Transgenic adenocarcinoma mouse prostate
ULBPs: HCMV glycoprotein UL16-binding protein family molecules
