# Supplementary figures and images for: MICA immune complex formed with alpha 3 domain-specific antibody activates human NK cells in a Fc-dependent manner
Source: J Immunother Cancer. 2019 Aug 6;7:207. doi: 10.1186/s40425-019-0687-9 (PMC6685158; doi:10.1186/s40425-019-0687-9)

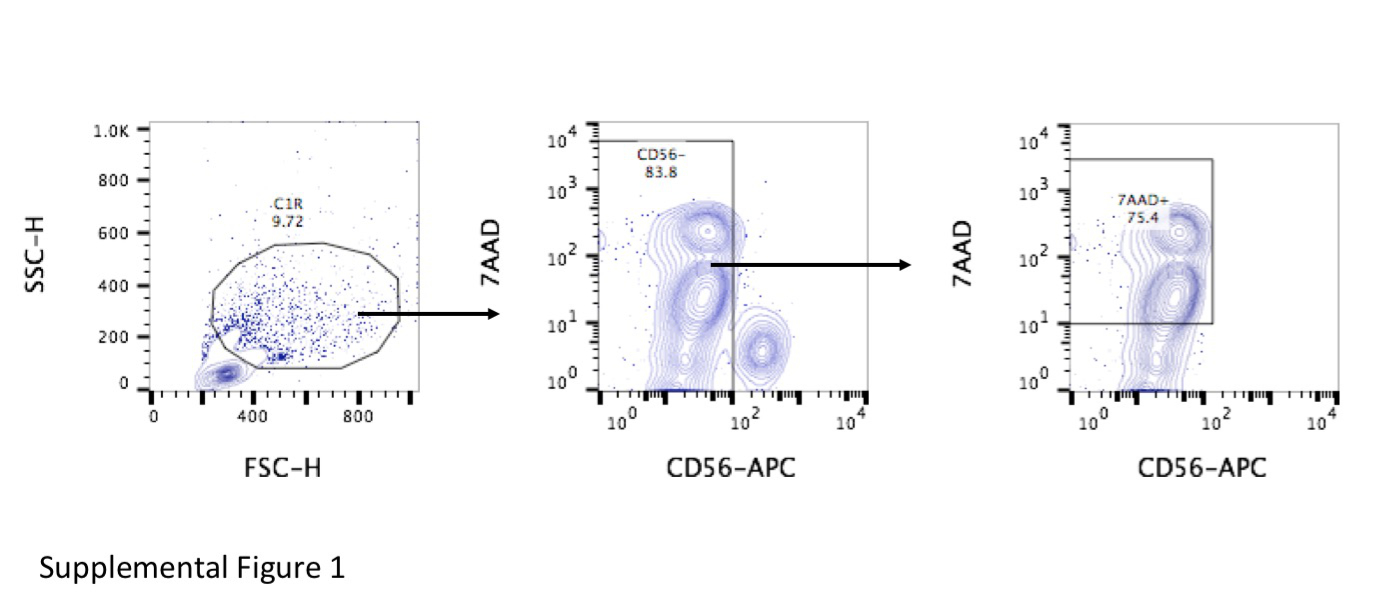

Supplement: Supplementary file 1 — Figure S1. FACS gating strategy for NK cytolytic activity assay. To analyze the NK cytolytic data acquired on FACSCalibur, target tumor cells were initially gated based on forward scatter (FSC) vs. side scatter (SSC). The CD56− target cell population was subsequently gated based on the dot plot of CD56-APC vs. 7-AAD to exclude contaminating NK cells; the 7-AAD+ target cell was finally gated within the CD56− gate to report target cell death as the percentage of 7-AAD+ cell population within the total CD56− cell population. (JPG 204 kb) [file 40425_2019_687_MOESM1_ESM.jpg]

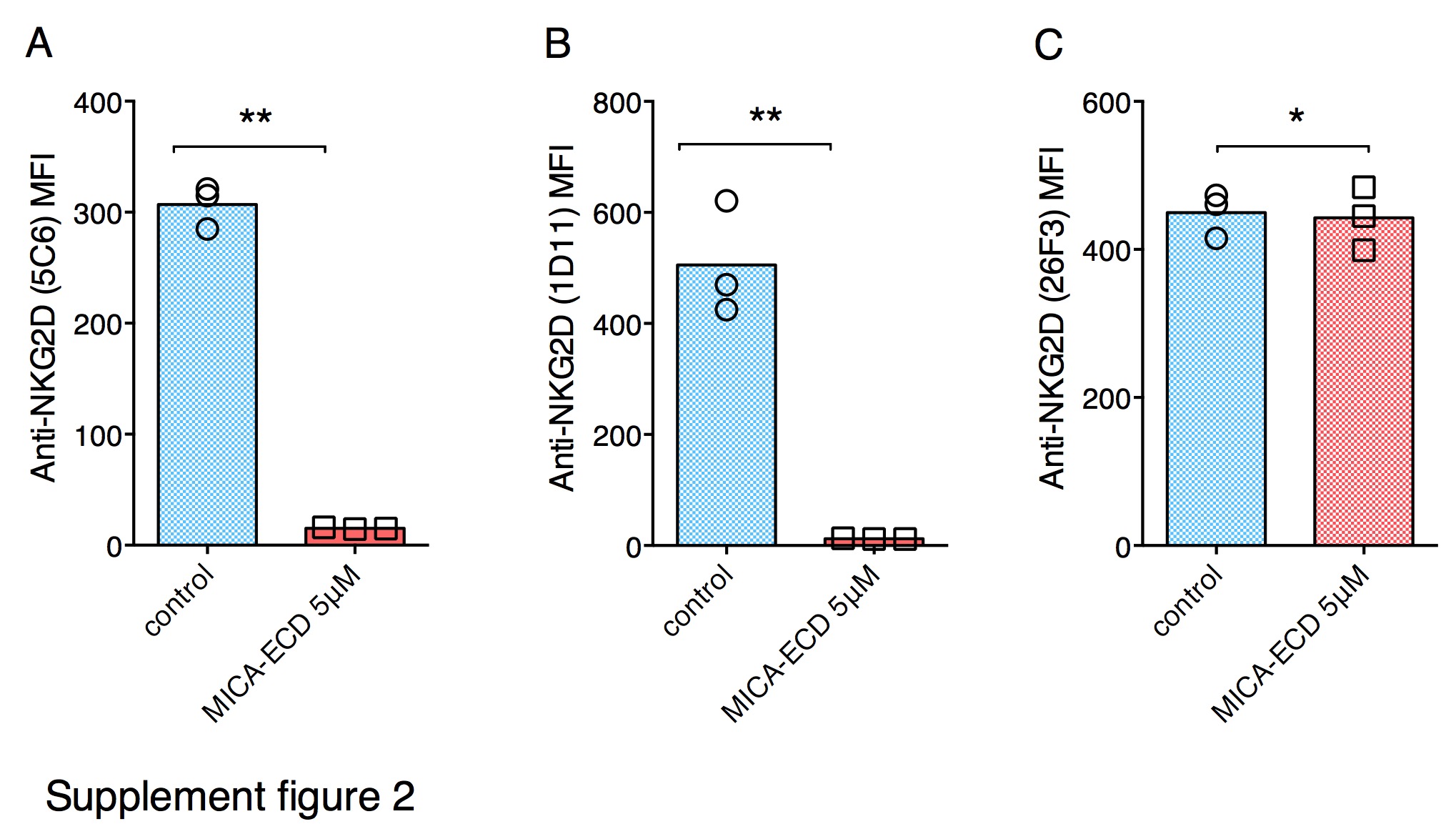

Supplement: Supplementary file 2 — Figure S2. The anti-NKG2D mAb clone 26F3 does not compete with MICA-ECD. NKG2D on NK cells was not detected by anti-NKG2D clones 5C6 (A) or 1D11 (B), but by 26F3 (C) following treatment with 5 μM MICA-ECD at 4 °C for 4 h. The experiment was conducted using NK cells isolated from three independent donors (error bar representing SD), and p-values were generated from unpaired t-test. *p > = 0.05; **p < 0.05. (JPG 336 kb) [file 40425_2019_687_MOESM2_ESM.jpg]

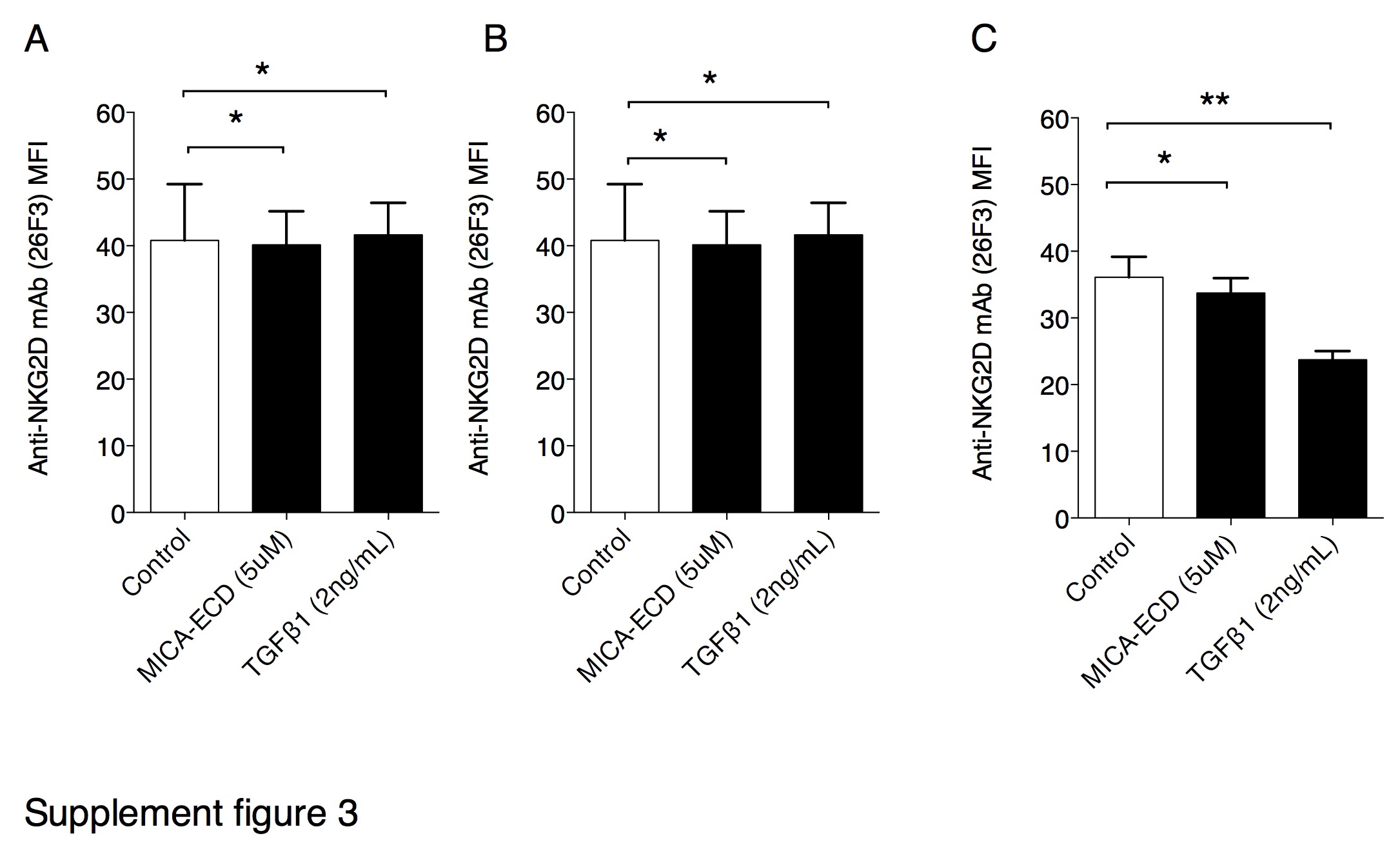

Supplement: Supplementary file 3 — Figure S3. TGFβ1 but not MICA-ECD down-regulates NKG2D on NK cells in the temporal analysis of NKG2D expression. NK cells from 3 healthy donors were treated with soluble MICA-ECD, TGFβ1 or no treatment, NKG2D expression was analyzed using non-competing anti-NKG2D antibody 26F3 on FACSCalibur at 4 (A), 8 (B) and 24 (C) hour time point. The NKG2D expression was indicated as the mean fluorescence intensity (MFI) of antibody stained cell population by flow cytometry analysis. The error bar represents SD, and the dataset is a representative of 2 independent experiments with p-values generated from unpaired t-test. *p > = 0.05; **p < 0.05. (JPG 227 kb) [file 40425_2019_687_MOESM3_ESM.jpg]

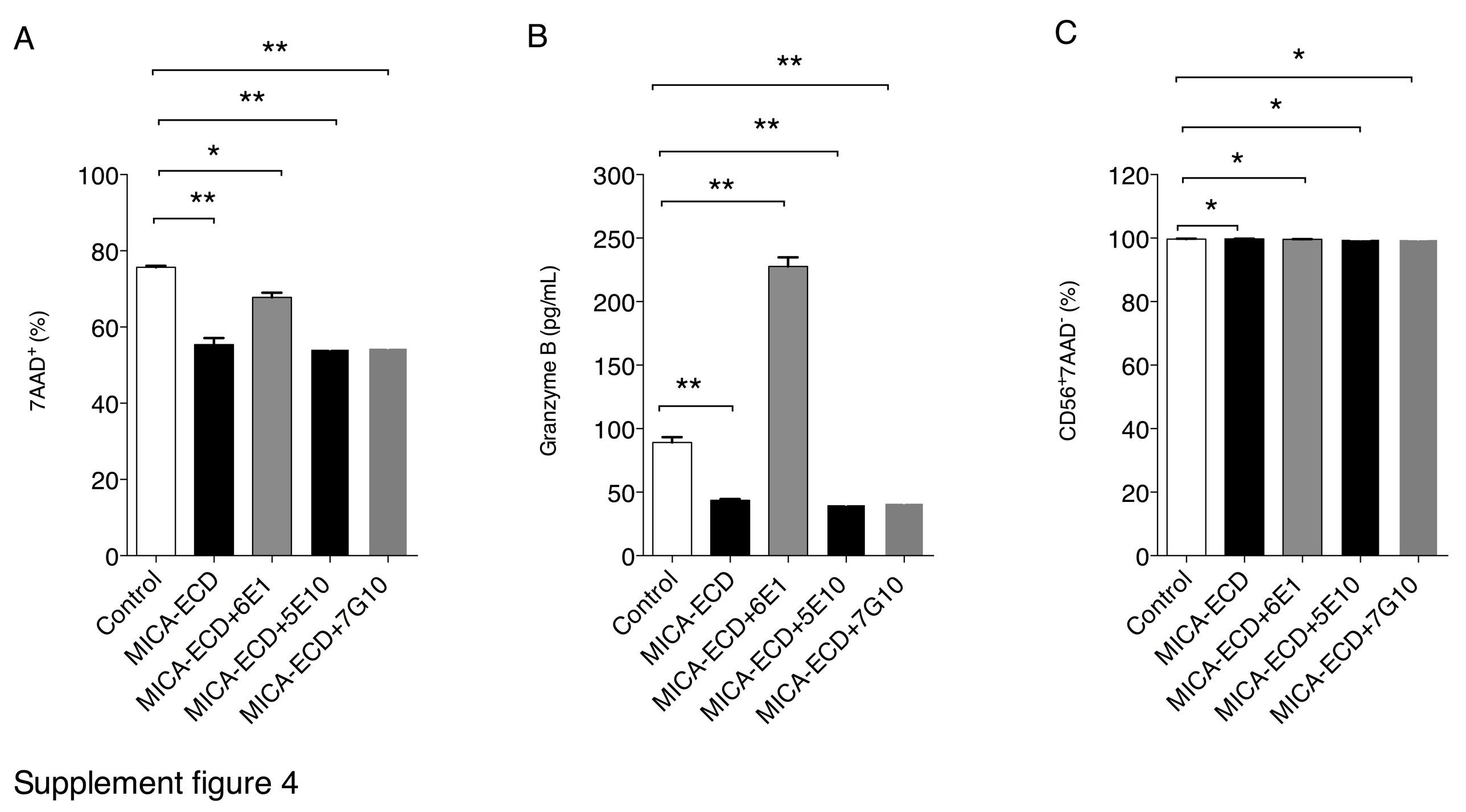

Supplement: Supplementary file 4 — Figure S4. The MICA immune complexes formed with α3-specific antibody 6E1 reverse MICA-ECD-mediated NK cell suppression and induced Granzyme B release. a, C1R-MICA*002 cell line killing experiment was conducted by co-culturing C1R-MICA*002 cell line with primary NK cells for 4 h. The NK cells were pretreated with MICA-ECD alone, MICA-ECD plus anti-MICA/B clones, 6E1 (MICA α3-specific) or 5E10 (MICA α1α2-specific) or 7G10 (MICA α1α2-specific) as preformed MICA-immune complexes, or no treatment. Each data point represents an average of 3 technical replicates with error bar representing SD, and the dataset is a representative of 3 independent experiments with p values generated from unpaired t test. b, Granzyme B release in the supernatants of C1R-MICA*002 cell line killing experiment was quantified by ELISA assay (Human Granzyme B DuoSet ELISA kit, R&D systems), each data point represents average of 3 technical replicates with error bar representing SD, and the dataset is a representative of 3 independent experiments with p-values generated from unpaired t-test. c, NK cell viability in the co-culture across all experiment groups were examined and the percentage of CD56+ 7AAD− NK cells in total NK cell population are shown. Each data point represents average of 3 technical replicates with error bar representing SD, and the dataset is a representative of 3 independent experiments with p-values generated from unpaired t-test. *p > = 0.05; **p < 0.05. (JPG 2307 kb) [file 40425_2019_687_MOESM4_ESM.jpg]

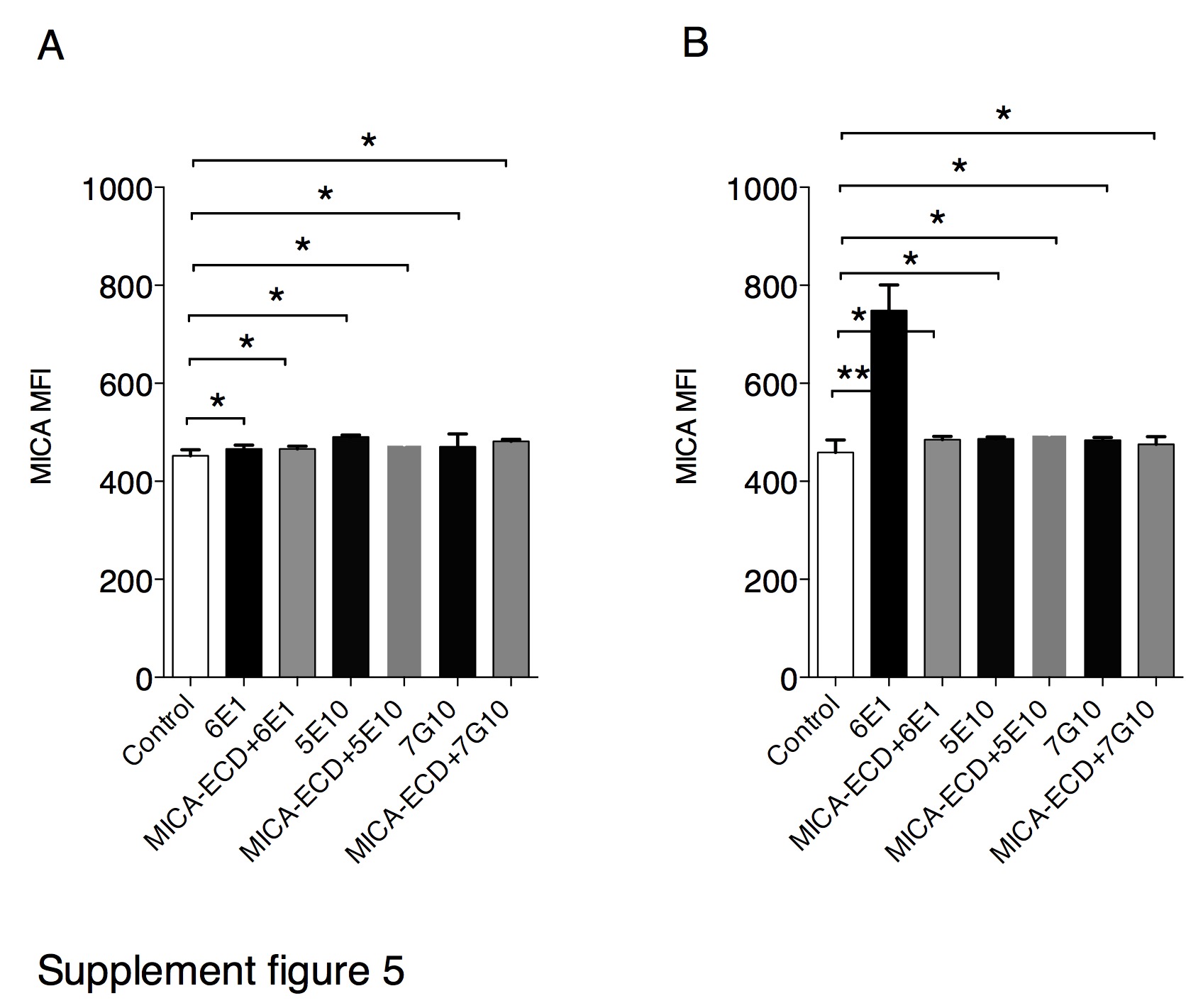

Supplement: Supplementary file 5 — Figure S5. MICA α3-specific antibody 6E1 but not MICA-immune complexes stabilizes cell surface MICA. C1R-MICA*002 cells were treated by control hIgG1, 6E1, 5E10, 7G10 or MICA immune complex preformed with each of the 3 MICA antibodies, and the MICA expression was captured by non-competing α1α2-specific anti-MICA antibody (clone 6D4, eBioscience). The samples were collected at 4 (A) and 8 (B) hour time point, each data point is an average of 3 technical replicates with error bar representing SD, and the dataset is a representative of 2 independent experiments with p-values generated from unpaired t-test. *p > = 0.05; **p < 0.05. (JPG 209 kb) [file 40425_2019_687_MOESM5_ESM.jpg]

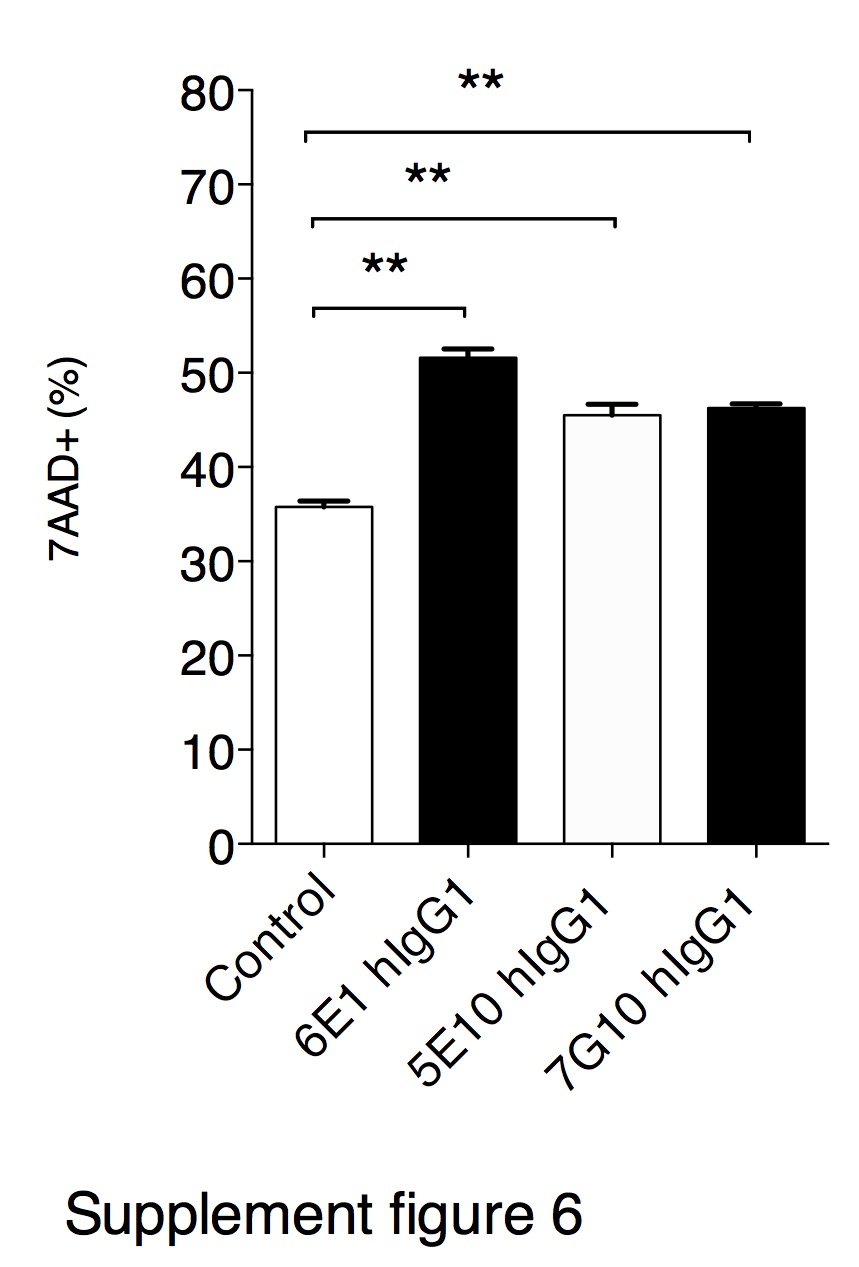

Supplement: Supplementary file 6 — Figure S6. MICA antibodies induce ADCC. C1R-MICA*002 cell line ADCC experiment was conducted by co-culturing C1R-MICA*002 cell line with primary NK cells (10 to 1 effector to target ratio) in the presence of 10 μg/mL control hIgG1 or anti-MICA antibodies, 6E1, 5E10 or 7G10 for 4 h, the data is the average of 3 technical replicates with error bar representing SD, and the dataset is a representative of 3 independent experiments with p-values generated from unpaired t-test. *p > = 0.05; **p < 0.05. (JPG 95 kb) [file 40425_2019_687_MOESM6_ESM.jpg]
