# Supplementary material for: MICA immune complex formed with alpha 3 domain-specific antibody activates human NK cells in a Fc-dependent manner
Source: J Immunother Cancer. 2019 Aug 6;7:207. doi: 10.1186/s40425-019-0687-9 (PMC6685158; doi:10.1186/s40425-019-0687-9)
Supplement: Supplementary file 7 — Table S1. Specificity and affinity of anti-MICA antibodies (DOCX 14 kb) [file 40425_2019_687_MOESM7_ESM.docx]

|  | **ELISA^1^** | **ELISA^1^** | **Biacore^2^** | **Biacore^2^** |
| --- | --- | --- | --- | --- |
| Clone | MICA*002 α3 | MICA*002-ECD | Kd (nM) | Kd (nM) |
|  |  |  | MICA*002α3 | MICA*002-ECD |
| 6E1 | + | + | 2.96 | n.d. |
| 5E10 | - | + | n.d. | 0.29 |
| 7G10 | - | + | n.d. | 0.79 |

Table S1. Specificity and affinity of anti-MICA antibodies

1, Enzyme-linked immunosorbent assay (ELISA) was used to determine the binding specificity of anti-MICA/B antibodies, 6E1, 5E10 or 7G10. Assay plate was coated with recombinant MICA*002 α3 or MICA*002-ECD, followed by anti-MICA/B antibody binding. The binding of anti-MICA/B antibody was detected by HRP-conjugated goat anti-mouse IgG Fc and TMB substrate. ELISA values >0.5 are designated with a plus sign, ELISA values <0.5 are designated with a minus sign. n.d. = not determined.

2, The binding kinetics of the anti-MICA/B antibodies were measured using surface plasmon resonance (SPR) on a Biacore T200 instrument (GE Healthcare). A CM5 sensorchip (GE Healthcare) was coated with anti-mouse Fc to capture mouse anti-MICA/B antibodies, 6E1, 5E10 or 7G10. The recombinant MICA*002 α3 or MICA*002-ECD was passed over. Antibody binding was measured to human recombinant MICA*002 α3 or MICA*002-ECD (His tagged, in-house). The data was analyzed using a 1:1 binding model. Several binding affinities (Kd) to MICA*002 α3 or MICA*002-ECD could not be determined by Biacore due to technical reasons.
